# Neuronal hyperexcitability in the ventral posterior thalamus of neuropathic rats: modality selective effects of pregabalin

**DOI:** 10.1152/jn.00237.2016

**Published:** 2016-04-20

**Authors:** Ryan Patel, Anthony H. Dickenson

**Affiliations:** Department of Neuroscience, Physiology and Pharmacology, University College London, London, United Kingdom

**Keywords:** in vivo electrophysiology, ventral posterolateral thalamus, spinal nerve ligation, wide dynamic range, nociceptive specific

## Abstract

Neuropathic pain represents a substantial clinical challenge; understanding the underlying neural mechanisms and back-translation of therapeutics could aid targeting of treatments more effectively. The ventral posterior thalamus (VP) is the major termination site for the spinothalamic tract and relays nociceptive activity to the somatosensory cortex; however, under neuropathic conditions, it is unclear how hyperexcitability of spinal neurons converges onto thalamic relays. This study aimed to identify neural substrates of hypersensitivity and the influence of pregabalin on central processing. In vivo electrophysiology was performed to record from VP wide dynamic range (WDR) and nociceptive-specific (NS) neurons in anesthetized spinal nerve-ligated (SNL), sham-operated, and naive rats. In neuropathic rats, WDR neurons had elevated evoked responses to low- and high-intensity punctate mechanical stimuli, dynamic brushing, and innocuous and noxious cooling, but less so to heat stimulation, of the receptive field. NS neurons in SNL rats also displayed increased responses to noxious punctate mechanical stimulation, dynamic brushing, noxious cooling, and noxious heat. Additionally, WDR, but not NS, neurons in SNL rats exhibited substantially higher rates of spontaneous firing, which may correlate with ongoing pain. The ratio of WDR-to-NS neurons was comparable between SNL and naive/sham groups, suggesting relatively few NS neurons gain sensitivity to low-intensity stimuli leading to a “WDR phenotype.” After neuropathy was induced, the proportion of cold-sensitive WDR and NS neurons increased, supporting the suggestion that changes in frequency-dependent firing and population coding underlie cold hypersensitivity. In SNL rats, pregabalin inhibited mechanical and heat responses but not cold-evoked or elevated spontaneous activity.

## Abstract (precis)

*Studies on brain mechanisms of neuropathic pain are lacking. This study characterizes the properties of rat ventral posterior thalamic wide dynamic range (WDR) and nociceptive-specific (NS) neurons, the latter of which are uncharacterized in a neuropathic state. We provide evidence of phenotypic changes in neuronal sensitivity that may underlie cold and brush hypersensitivity, and that WDR neurons, and not NS neurons, encode hypersensitivity to low-intensity stimuli. Pregabalin reversed neuronal hyperexcitability in spinal nerve-ligated rats in a modality-selective manner*.

## NEW & NOTEWORTHY

*Studies on brain mechanisms of neuropathic pain are lacking. This study characterizes the properties of rat ventral posterior thalamic wide dynamic range (WDR) and nociceptive-specific (NS) neurons, the latter of which are uncharacterized in a neuropathic state. We provide evidence of phenotypic changes in neuronal sensitivity that may underlie cold and brush hypersensitivity, and that WDR neurons, and not NS neurons, encode hypersensitivity to low-intensity stimuli. Pregabalin reversed neuronal hyperexcitability in spinal nerve-ligated rats in a modality-selective manner*.

the thalamus integrates multiple sensory pathways including facets of nociception and is the termination site of the spinothalamic tract (STT). Nuclei of the lateral pathway, such as the ventral posterior (VP), largely project to the somatosensory cortex (S1 and S2), are somatotopically organized and typically associated with sensory-discriminatory aspects of pain, whereas medical nuclei, including the mediodorsal and intralaminar nuclei, project to the anterior cingulate cortex and insula and are concerned with affective and motivational components of pain. Patients undergoing stereotaxic procedures provide the unique opportunity to obtain electrophysiological recordings from the human thalamus and have the advantage of gaining qualitative feedback upon stimulation. From these studies it is evident that neurons within the ventral caudal region can encode intensity of peripherally applied stimuli. Furthermore, increasing the intensity of electrical stimulation within the thalamus correlates positively with intensity of sensation and can evoke pain, thermal sensations (both warm and cold), nonpainful paraesthesia, and mechanical sensations (Davis et al. 1999; Lenz et al. 1993; Ohara et al. 2004).

Both lamina I and lamina V in the dorsal horn have projections to the ventral posterolateral nucleus (VPL) (Willis et al. 2001), and neurons within the STT–VP–S1-S2 pathway are predominantly wide dynamic range (WDR). This is in marked contrast to neurons within medial pathways, which are almost exclusively nociceptive specific (NS) (Whitt et al. 2013). At the spinal level, WDR neurons discriminate between small differences in stimulus intensity, whereas NS neurons have reduced capacity to do so (Dubner et al. 1989; Maixner et al. 1986), and in parallel animal and human studies this fine-tuned neuronal coding in rats correlated with psychophysical performance to the same stimuli in healthy human volunteers under normal conditions (Sikandar et al. 2013) and in surrogate models of central sensitization (O′Neill et al. 2015). These studies support the importance of WDR neurons and the STT–VP–S1-S2 pathway to sensory discrimination; hence, we have focused on characterizing the neurophysiological properties of VP WDR and NS neurons in normal and neuropathic conditions given their proposed nociceptive roles.

Stimulus-response relationships of VP WDR and NS neurons in uninjured conditions have been described previously in various species including in the rat, cat, raccoon, and primates (Apkarian and Shi 1994; Chung et al. 1986; Guilbaud et al. 1980; Simone et al. 1993; Yokota et al. 1988). To date, their properties in neuropathic animals have been less well characterized, in particular with respect to cold sensitivity, disturbances of which are prominent in chemotherapy-induced neuropathy and other neuropathic conditions (Maier et al. 2010). Electrophysiological recordings obtained from models of diabetic neuropathy, spinal cord injury, sciatic nerve ligation, and rheumatoid arthritis have all identified elevated spontaneous and evoked activity, although these studies have largely focused on WDR neurons and responses to mechanical stimulation (Fischer et al. 2009; Gautron and Guilbaud 1982; Guilbaud et al. 1990; Hains et al. 2005; Miki et al. 2000; Vos et al. 2000).

The present study aims to examine thalamic mechanisms of hypersensitivity in the spinal nerve ligation (SNL) model by utilizing in vivo electrophysiology. Furthermore, we investigate mechanisms by which pregabalin provides relief to evoked and ongoing pain. Pregabalin is an integral part of frontline therapy for various neuropathies of peripheral and central origin, with NNT (number needed to treat) values ranging from 6 to 9 (Finnerup et al. 2015; Moore et al. 2009). In neuropathic animals, gabapentinoids can abolish behavioral hypersensitivity measured by changes in withdrawal threshold (Field et al. 1997) and attenuate responses of spinal neurons at higher intensities of stimulation (Suzuki et al. 2005). However, the neural mechanisms by which gabapentinoids provide relief from ongoing pain are poorly understood, and nothing is known about their effects on sensory processing in the brain.

## MATERIALS AND METHODS

### 

#### Animals.

Naive male Sprague-Dawley rats or sham spinal nerve-ligated (SNL) rats (260–315 g) were used for electrophysiological experiments (Biological Services, University College London, UK). Animals were group housed (maximum of 5) on a 12:12-h light-dark cycle, and food and water were available ad libitum. Temperature (20–22°C) and humidity (55–65%) of holding rooms were closely regulated. All procedures described were approved by the UK Home Office, adhered to the Animals (Scientific Procedures) Act 1986, and were in accordance with International Association for the Study of Pain ethics guidelines (Zimmermann 1983).

#### SNL surgery.

Unilateral SNL surgery was performed as previously described (Patel et al. 2014a). Rats (140–150 g at the time of surgery) were maintained under 2% (vol/vol) isoflurane anesthesia delivered in a 3:2 ratio of nitrous oxide and oxygen. Under aseptic conditions, a paraspinal incision was made and the tail muscle excised. Part of the L5 transverse process was removed to expose the left L5 and L6 spinal nerves, which were then isolated with a glass nerve hook (Ski-Ry, London, UK) and ligated with a nonabsorbable 6-0 braided silk thread proximal to the formation of the sciatic nerve. The surrounding skin and muscle was closed with absorbable 3-0 sutures. Sham surgery was performed in an identical manner, omitting the ligation step. All rats groomed normally and gained weight in the following days postsurgery. All electrophysiological experiments were performed between postoperative *days 14* and *18*.

#### In vivo electrophysiology.

**Fig. 1.**
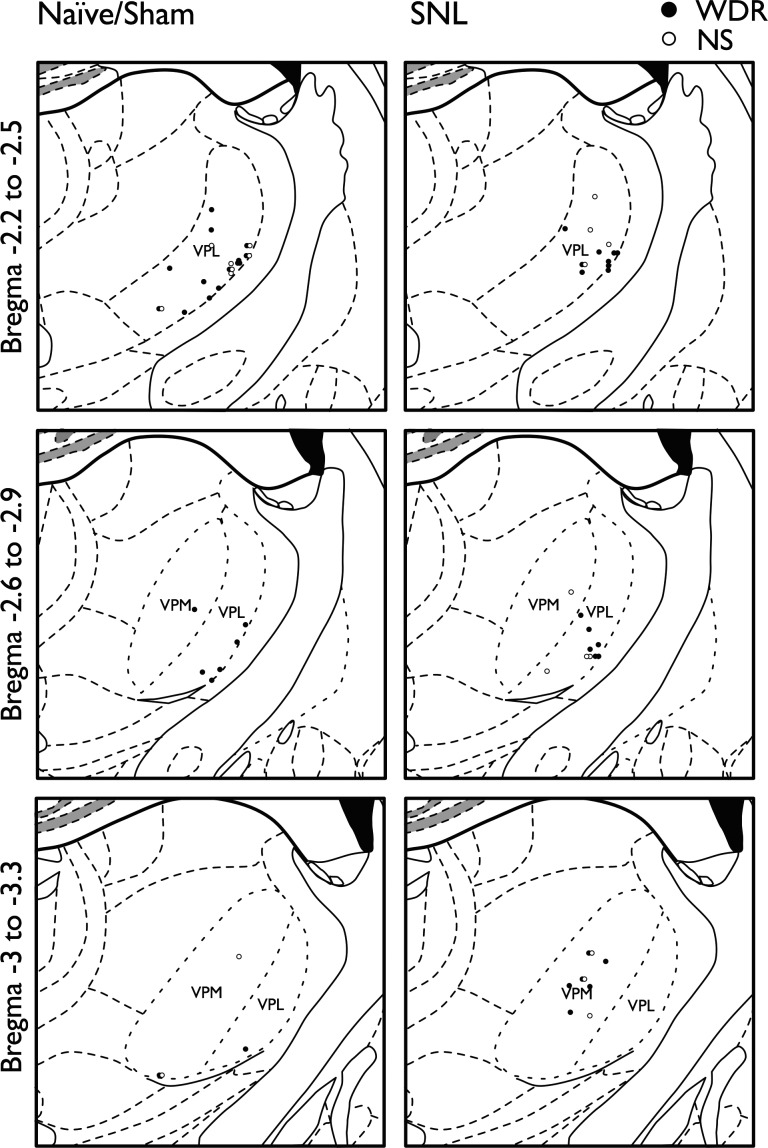
Recording sites within the ventral posteromedial (VPM) and ventral posterolateral (VPL) nuclei of the thalamus in naive/sham and spinal nerve-ligated (SNL) rats. Filled circles represent wide dynamic range (WDR) neurons; open circles represent nociceptive-specific (NS) neurons.

Animals were initially anesthetized with 3.5% (vol/vol) isoflurane delivered in a 3:2 ratio of nitrous oxide and oxygen. Once the animals were areflexic, a tracheotomy was performed and rats were subsequently maintained on 1.5% (vol/vol) isoflurane for the remainder of the experiment. Rats were secured in a stereotaxic frame, and after the skull was exposed, coordinates for the right ventral posterolateral nucleus (VPL) of the thalamus (contralateral to injury) were calculated in relation to bregma (2.28 mm caudal, 3.2 mm lateral; Watson and Paxinos 2006). A small craniotomy was performed with a high-speed surgical microdrill, and the overlying dura was removed. Extracellular recordings were made from ventral posteromedial (VPM) and VPL thalamic neurons with receptive fields on the hind toes of the left paw (see Fig. 1 for recording sites based on coordinates) using 0.127-mm-diameter, 2-MΩ parylene-coated tungsten electrodes (A-M Systems, Sequim, WA). Neurons were selected on the basis of responses to brush, noxious punctate mechanical stimulation, and noxious thermal stimulation of the receptive field. The receptive field was stimulated using a range of natural stimuli (brush; von Frey filaments: 2, 8, 15, 26, and 60 g; and heat: 35, 42, 45, and 48°C) applied over a period of 10 s per stimulus. The heat stimulus was applied with a constant water jet onto the center of the receptive field. Acetone and ethyl chloride (100 μl; Miller Medical Supplies, Newport, UK) were applied as an evaporative innocuous cooling and noxious cooling stimulus, respectively. Evoked responses to room temperature water (25°C) were minimal, or frequently completely absent, and were subtracted from acetone- and ethyl chloride-evoked responses to control for any concomitant mechanical stimulation during application. Stimuli were applied starting with the lowest intensity stimulus with ∼30–40 s between stimuli in the following order: brush, von Frey, cold, heat. Data were captured and analyzed by a Cambridge Electronic Design 1401 interface coupled to a computer with Spike2 software (CED, Cambridge, UK) with rate functions. Spike sorting was performed post hoc with Spike2 using waveform analysis followed by principal component analysis. Stimulus-evoked neuronal responses were determined by subtracting total spontaneous neuronal activity in the 10-s period immediately preceding stimulation. Spontaneous firing of individual neurons (number of spikes per second) is expressed as the mean of these 10-s periods.

At a subset of recording sites (1 per rat), pharmacological studies were performed. After three baseline responses to natural stimuli (applied in the order described above; data were averaged to give control values), rats were injected with 0.9% saline subcutaneously (1 ml/kg) and neuronal responses were characterized 30 min postdosing. Rats were subsequently injected with 10 mg/kg pregabalin (gift from Pfizer; vehicle: 0.9% saline) subcutaneously (1 ml/kg), and neuronal responses were characterized 30 and 50 min postdosing; peak change from baseline is plotted. The dose of pregabalin was chosen on the basis of reversal of behavioral hypersensitivity without sedative or motor side effects (Abbadie et al. 2010). In addition, no further dose-dependent inhibitions are observed on spinal neuronal responses above this dose (Bannister et al. 2011).

A total of 10 naive, 9 sham, and 24 SNL rats were used in this study. The number of recording sites ranged from one to four per rat; one to three neurons were characterized at each site. As required by the appropriate authorities, on ethical grounds, all electrophysiological procedures were nonrecovery; at the end of the experiments, rats were terminally anesthetized with isoflurane.

#### Statistics.

Statistical analyses were performed using SPSS (version 22; IBM, Armonk, NY). Heat and mechanical coding of neurons were compared with a two-way ANOVA or two-way repeated-measures (RM) ANOVA, followed by a Bonferroni post hoc test for paired comparisons. Where appropriate, sphericity was tested using Mauchly's test; the Greenhouse-Geisser correction was applied if violated. Cold, brush, and spontaneous firing were compared with either a two-tailed paired Student's *t*-test or two-tailed unpaired Student's *t*-test (Welch's correction was applied if Levene's test of equal variances was violated). Frequency distributions were compared with a two-tailed Fisher's exact test. Group sizes were determined by a priori calculations (α = 0.05, 1 − β = 0.8). All data represent means ± SE.

## RESULTS

### 

#### Spontaneous and evoked activity of WDR neurons of the VP nuclei is elevated in SNL rats.

**Fig. 2.**
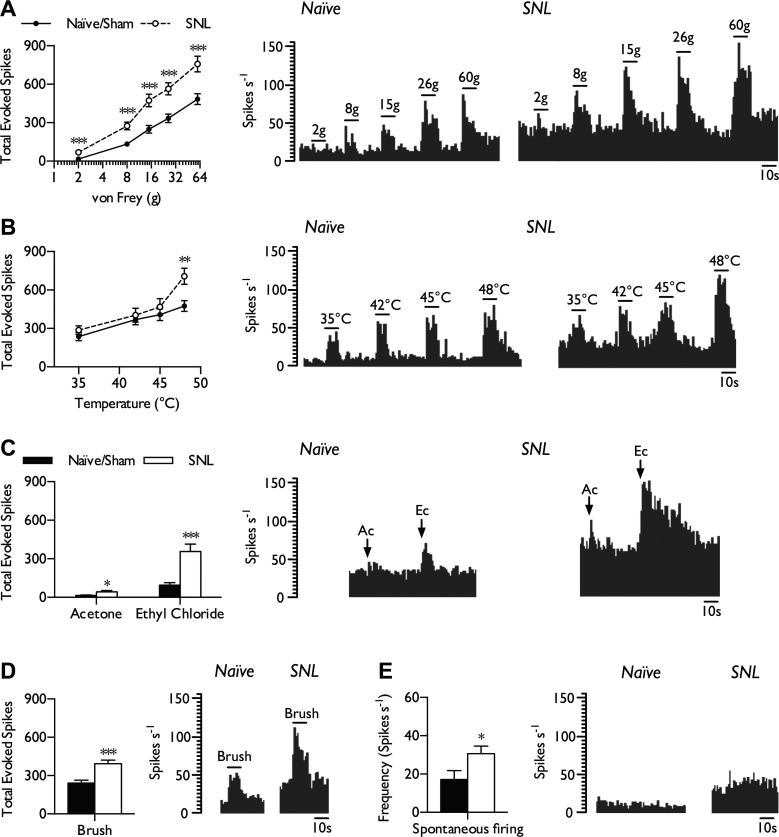
Hyperexcitability of WDR neurons to low- and high-intensity stimuli in SNL rats. In neuropathic conditions, WDR neurons exhibited elevated responses to punctate mechanical stimulation (*A*), noxious heat (*B*), innocuous (acetone) and noxious (ethyl chloride) evaporative cooling (*C*), dynamic brushing (*D*), and spontaneous firing (*E*). Histogram traces represent typical single-unit responses. Naive/sham, *n* = 31 neurons from 18 rats; SNL, *n* = 35 neurons from 16 rats. All evoked responses represent total spikes over 10 s from start of stimulation. Data represent means ± SE. *P < 0.05; **P < 0.01; ***P < 0.001. Ac, acetone; Ec, ethyl chloride.

Our personal observation is that the majority of mechanically evoked activity within the VP nuclei is innocuous (i.e., brush evoked, onset/offset firing, noncoding to intensity) or proprioceptive (flexing of joints). This corresponds well with a previous study in the squirrel monkey, which identified 90% of neurons within this region as nonnociceptive, with the remaining population being of either WDR or NS phenotype (Apkarian and Shi 1994), and would fit with a large dorsal column projection to this area. WDR neurons were categorized according to responses to dynamic brushing, low- and suprathreshold punctate mechanical stimulation, and noxious heat stimulation of the receptive field. Within the VP nuclei, WDR neurons were able to encode intensity of stimulus ranging from low to high threshold across a range of modalities (mechanical, heat, and cold). WDR neurons in naive and sham rats displayed similar coding properties and thus were pooled for analysis (naive, *n* = 16 from 10 rats; sham, *n* = 15 from 9 rats). In neuropathic rats, we found strong evidence that WDR responses to both low (2 and 8 g)- and high-intensity mechanical stimuli (15, 26, and 60 g) were substantially increased compared with responses in the naive/sham group (Fig. 2*A*; 2-way ANOVA, *P* = 0.000015, *F*_1,320_ = 60.02). In contrast, coding to innocuous (35°C) and perithreshold (42–45°C) heat stimulation was comparable between both groups; heat hypersensitivity in SNL rats was only evident at noxious levels of stimulation (48°C; Fig. 2*B*; 2-way ANOVA, *P* = 0.0037, *F*_1,256_ = 8.592). Acetone and ethyl chloride were applied as evaporative cooling stimuli, previously shown to reliably and reproductively deliver an innocuous and noxious cold stimulus, respectively, based on skin temperature recordings and electromyography (Leith et al. 2010). Both innocuous and noxious cold temperatures evoked greater neuronal responses in SNL rats compared with controls (Fig. 2*C*; unpaired *t*-test with Welch's correction, acetone: *P* = 0.015; ethyl chloride: *P* = 0.0001). In addition, all WDR neurons characterized were sensitive to dynamic brushing of the receptive field (>5 spikes/s); responses to brushing were also higher in SNL rats compared with naive/sham rats (Fig. 2*D*; unpaired *t*-test, *P* = 0.000145). The majority of naive/sham WDR neurons (25/31) exhibited spontaneous firing ranging from 1.4 to 96.8 spikes/s, whereas almost all SNL neurons (34/35) exhibited spontaneous firing ranging from 3.82 to 91.2 spikes/s; the remaining neurons exhibited low levels of activity (<1 spike/s). Overall, WDR neurons in SNL rats had higher rates of spontaneous firing (Fig. 2*E*; unpaired *t*-test, *P* = 0.0302).

#### Evoked activity of NS neurons of the VP nuclei is elevated in SNL rats.

**Fig. 3.**
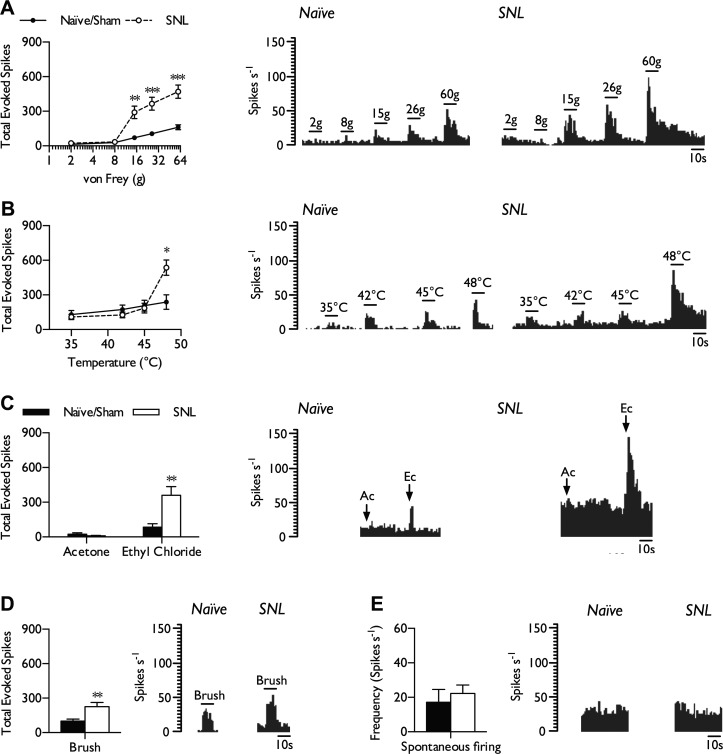
Hyperexcitability of NS neurons to high-intensity stimuli in SNL rats. In neuropathic conditions, NS neurons exhibited elevated responses to punctate mechanical stimulation (*A*), noxious heat (*B*), noxious (ethyl chloride) evaporative cooling (*C*), and dynamic brushing (*D*) but no change in spontaneous firing (*E*). Histogram traces represent typical single-unit responses. Naive/sham, *n* = 14 neurons from 9 rats; SNL, *n* = 19 neurons from 10 rats. All evoked responses represent total spikes over 10 s from start of stimulation. Data represent means ± SE. *P < 0.05; **P < 0.01; ***P <0.001.

Like WDR neurons, NS neurons had the capacity to encode intensity of stimulus, but did so selectively within the noxious range and with reduced ability to differentiate between intensities. In general, evoked responses of NS neurons were smaller in magnitude compared with those of WDR neurons. As observed with WDR neurons, NS neurons in naive and sham rats also displayed similar coding properties and were pooled for analysis (naive, *n* = 6 from 4 rats; sham, *n* = 8 from 5 rats). NS neurons exhibited minimal or no evoked firing (<5 spikes/s) to low-threshold punctate mechanical stimuli (2 and 8 g) and responded in an intensity-dependent manner to more noxious intensities (15, 26, and 60 g). In neuropathic rats, NS neurons were more responsive to noxious punctate mechanical stimulation of the receptive field while continuing to respond minimally to low-threshold stimuli (Fig. 3*A*; 2-way ANOVA, *P* = 0.000015, *F*_1,155_ = 46.39). As observed in the WDR population, heat hypersensitivity in SNL rats was only evident at noxious levels of stimulation (48°C; Fig. 3*B*; 2-way ANOVA, *P* = 0.045, *F*_1,124_ = 4.1). NS neurons had minimal innocuous cold sensitivity in the absence of and after nerve injury (unpaired *t*-test with Welch's correction, *P* = 0.297), although noxious cold-evoked activity was evident in naive/sham rats, and these responses were elevated after peripheral nerve injury (Fig. 3*C*; unpaired *t*-test with Welch's correction, *P* = 0.00243). NS neurons in SNL rats also had increased responses to dynamic brushing of the receptive field (Fig. 3*D*; unpaired *t*-test, *P* = 0.00856). Spontaneous activity ranged from 1.53 to 94.2 spikes/s (12/14 neurons) in naive/sham rats and from 1.3 to 66.7 spikes/s in SNL rats (18/19). Overall, we found little evidence for altered spontaneous firing rates of NS neurons in SNL rats compared with the naive/sham group (Fig. 3*E*; unpaired *t*-test, *P* = 0.589).

#### Proportion of cold-sensitive WDR and NS neurons increases after SNL.

**Fig. 4.**
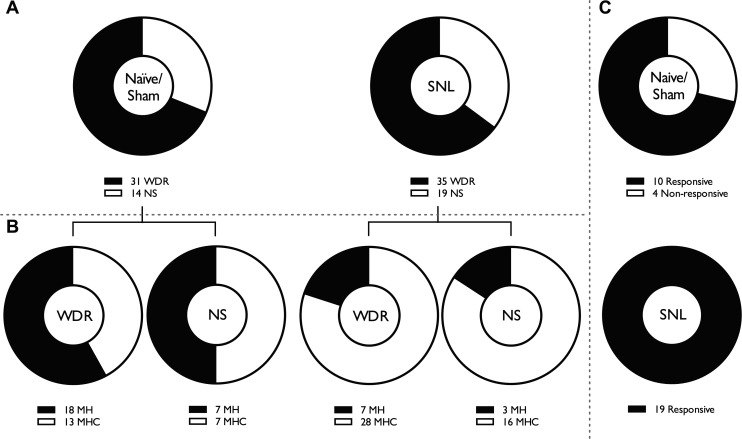
Characteristics of WDR and NS neurons are altered in neuropathic conditions. Similar numbers of WDR and NS neurons were found in naive/sham and SNL rats (*A*). In SNL rats, an increased proportion of WDR and NS neurons were responsive to noxious mechanical, heat, and cold stimulation (MHC) compared with noxious mechanical and heat stimulation (MH) (*B*). A subset of NS neurons may gain sensitivity to dynamic brushing after nerve injury (*C*).

Previous studies in primates reflect a preponderance of WDR neurons in the VP nuclei, outnumbering NS neurons by ∼4:1 (Apkarian and Shi 1994; Chung et al. 1986; Kenshalo et al. 1980). Although NS neurons were more prevalent in the rat VP, WDR neurons were encountered more frequently compared with NS neurons, and both naive/sham and SNL groups appeared to have similar proportions of WDR to NS neurons (Fig. 4*A*; Fisher's exact test, *P* = 0.831). All WDR and NS neurons characterized were responsive to noxious mechanical and heat (MH) stimulation of the receptive field, and some additionally responded to noxious cold stimulation (MHC). In naive/sham rats, 42% of WDR neurons were classified as MHC, although in SNL rats this increased to 80% (Fisher's exact test, *P* = 0.0022). We found weak evidence for similar changes among NS neurons, where 50% were MHC in naive/sham rats, and this rose to 84% in SNL rats (Fig. 4*B*; Fisher's exact test, *P* = 0.0569). In addition, in naive/sham rats, the majority of NS neurons exhibited brush-evoked responses (71%); however, brush sensitivity was evident in all NS neurons characterized in SNL rats (Fig. 4*C*; Fishers exact test, *P* = 0.0245).

#### Pregabalin inhibits mechanically evoked and brush- and heat-evoked WDR neuronal responses but not cold-evoked and spontaneous firing in SNL rats.

**Fig. 5.**
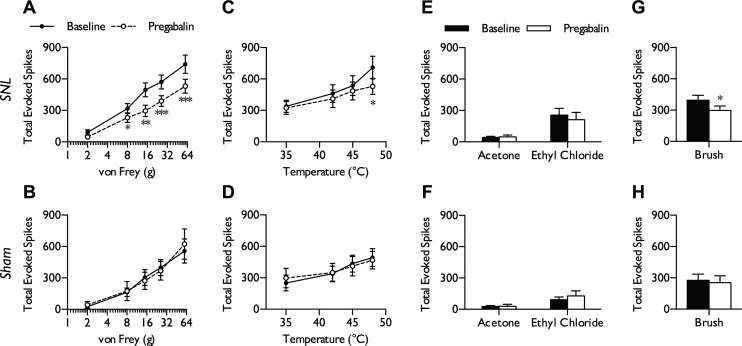
Pregabalin reduces evoked WDR neuronal responses in SNL rats. Neuronal responses to punctate mechanical (*A* and *B*), heat (*C* and *D*), cold (*E* and *F*), and brush stimuli (*G* and *H*) in SNL and sham rats pre- and post-pregabalin dosing. SNL, *n* = 15 neurons from 8 rats (*A*, *C*, *E*, and *G*); sham, *n* = 8 neurons from 8 rats (*B*, *D*, *F*, and *H*). Data represent means ± SE. *P < 0.05; **P < 0.01; ***P < 0.001.

**Fig. 6.**
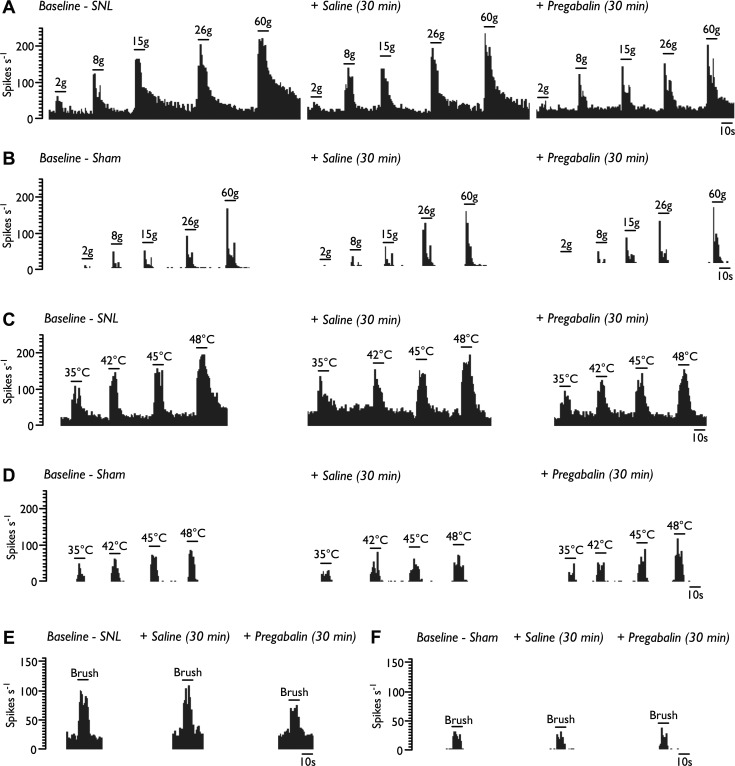
Single-unit histogram traces of WDR neurons pre- and post-pregabalin dosing. Pregabalin reduced neuronal responses to punctate mechanical stimulation in SNL rats (*A*) but not in sham rats (*B*). Pregabalin selectively reduced noxious heat-evoked responses in SNL rats (*C*) but had no effect in sham rats (*D*). Pregabalin reduced firing frequency to dynamic brushing in SNL rats (*E*) but not in sham rats (*F*).

Evoked and spontaneous firing rates were stable and reproducible over extended periods of time. Control baseline responses were calculated as the mean of three trials. Rats were then injected subcutaneously with 0.9% saline (1 ml/kg) and evoked responses characterized 30 min postdosing; evoked responses of both WDR and NS neurons, in SNL and sham rats, were comparable to baseline responses (data not shown). Rats were subsequently injected subcutaneously with 10 mg/kg pregabalin. In SNL rats, pregabalin inhibited WDR responses to both low- and high-intensity punctate mechanical stimulation of the receptive field (Figs. 5*A* and 6*A*; 2-way RM ANOVA, *P* = 0.00033, *F*_1,14_ = 22.225) and was without effect in sham-operated rats (Figs. 5*B* and 6*B*; 2-way RM ANOVA, *P* = 0.897, *F*_1,7_ = 0.018). Pregabalin inhibited heat-evoked responses in SNL rats selectively at noxious levels of intensity (48°C; Figs. 5*C* and 6*C*; 2-way RM ANOVA, *P* = 0.041, *F*_1,14_ = 5.047) and again was without effect in sham rats (Figs. 5*D* and 6*D*; 2-way RM ANOVA, *P* = 0.924, *F*_1,7_ = 0.01). Innocuous and noxious cold-evoked responses were unaffected by pregabalin in both SNL and sham rats (Fig. 5, *E* and *F*; paired *t*-test, SNL: acetone *P* = 0.530, ethyl chloride *P* = 0.759; sham: acetone *P* = 0.948, ethyl chloride *P* = 0.267). Brush-evoked responses were reduced by pregabalin in SNL (Figs. 5*G* and 6*E*; paired *t*-test, *P* = 0.021) but not sham-operated rats (Figs. 5*H* and 6*F*; paired *t*-test, *P* = 0.623). Interestingly, the elevated spontaneous WDR activity observed in neuropathic conditions was not inhibited by pregabalin (baseline: 27.6 ± 4.94 spikes/s, pregabalin: 27.8 ± 5.77 spikes/s; paired *t*-test, *P* = 0.951); spontaneous firing in sham rats was also unaffected (baseline: 6.09 ± 1.15 spikes/s, pregabalin: 3.86 ± 1.27 spikes/s; paired *t*-test, *P* = 0.089).

#### Pregabalin inhibits mechanically evoked and brush-evoked NS neuronal responses in SNL rats.

**Fig. 7.**
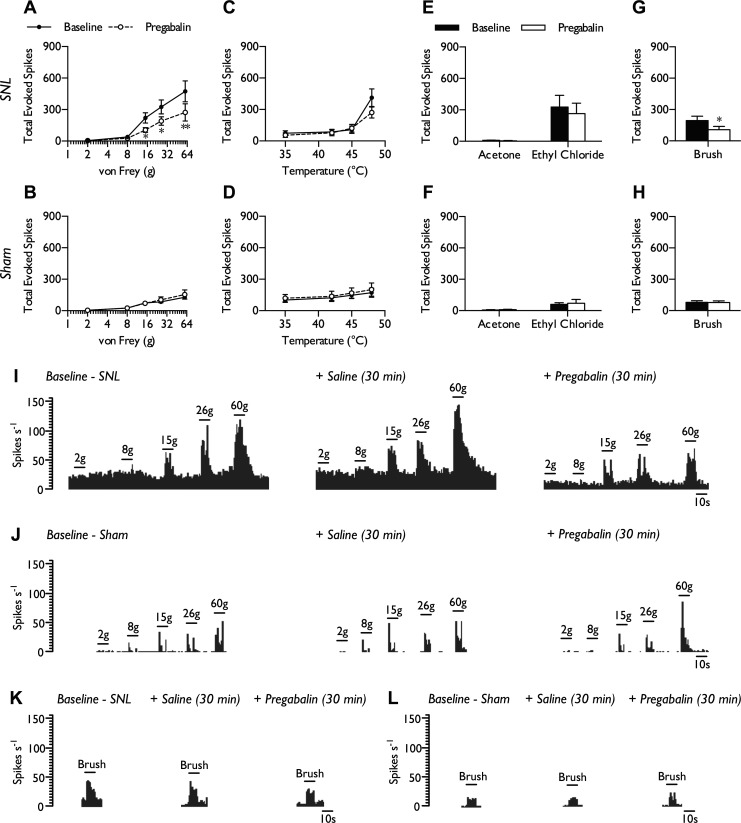
Pregabalin reduces evoked NS neuronal responses in SNL rats. Neuronal responses to punctate mechanical (*A* and *B*), heat (*C* and *D*), cold (*E* and *F*), and brush stimuli (*G* and *H*) in SNL and sham rats pre- and post-pregabalin dosing. SNL, *n* = 8 neurons from 5 rats(*A*, *C*, *E*, and *G*); sham, *n* = 7 neurons from 4 rats (*B*, *D*, *F*, and *H*). Data represent means ± SE. *P < 0.05; **P < 0.01. Single-unit histogram traces of NS neurons pre- and post-pregabalin dosing show that pregabalin reduced neuronal responses to punctate mechanical stimulation in SNL rats (*I*) but not in sham rats (*J*). Pregabalin also reduced firing frequency to dynamic brushing in SNL rats (*K*) but not in sham rats (*L*).

Similarly in NS neurons, pregabalin exhibited inhibitory activity dependent on pathophysiological state. Pregabalin inhibited neuronal responses to punctate mechanical stimulation (15, 26, and 60 g) in SNL (Fig. 7, *A* and *I*; 2-way RM ANOVA, *P* = 0.00831, *F*_1,7_ = 13.236) but not sham-operated rats (Fig. 7, *B* and *J*; 2-way RM ANOVA, *P* = 0.486, *F*_1,6_ = 0.552). In contrast to WDR neurons in neuropathic rats, NS responses to noxious heat stimulation (48°C) were not reduced by pregabalin (Fig. 7*C*; 2-way RM ANOVA, *P* = 0.184, *F*_1,7_ = 2.172), which again had no effect on the coding properties of sham NS neurons (Fig. 7*D*; 2-way RM ANOVA, *P* = 0.486, *F*_1,6_ = 0.475). As observed in WDR neurons, cold-evoked responses were conserved postdosing in both SNL (Fig. 7*E*; paired *t*-test, acetone *P* = 0.213, ethyl chloride *P* = 0.129) and sham rats (Fig. 7*F*; paired *t*-test, acetone *P* = 0.481, ethyl chloride *P* = 0.754). In addition, brush-evoked responses were inhibited by pregabalin in SNL rats (Fig. 7, *G* and *K*; paired *t*-test, *P* = 0.028), and this effect was absent in sham rats (Fig. 7, *H* and *L*; paired *t*-test, *P* = 0.898). As described above, the spontaneous firing rate of NS neurons was comparable between normal and neuropathic states. Similarly to that of WDR neurons, spontaneous firing of NS neurons was not reduced by pregabalin in both neuropathic (baseline: 12.5 ± 4.37 spikes/s, pregabalin: 9.34 ± 3.43 spikes/s; paired *t*-test, *P* = 0.317) and sham conditions (baseline: 5.87 ± 4.16 spikes/s, pregabalin: 1.82 ± 0.53 spikes/s; paired *t*-test, *P* = 0.373).

## DISCUSSION

In this article, we describe thalamic mechanisms of hypersensitivity in SNL rats. These data demonstrate hyperexcitability of WDR and NS neurons to brush, punctate mechanical, cold, and heat stimuli. To our knowledge, this study provides the first evidence of a gain in brush sensitivity of NS neurons in the VP nuclei in SNL rats, altered population coding to cold temperatures, unaltered levels of spontaneous activity in NS neurons, and modality-selective attenuation of evoked hypersensitivity but not aberrant spontaneous activity by pregabalin.

After a peripheral nerve injury, dorsal horn and gracile nucleus neurons do not display marked changes in frequency-dependent firing to mechanical, cold, or heat stimuli (Chapman et al. 1998; Palecek et al. 1992; Patel et al. 2014a; Patel et al. 2014b; Suzuki and Dickenson 2002). Although seemingly contradictory to behavioral hypersensitivity, this paradox could be explained by the denervation and loss of input as a result of nerve injury coupled with central sensitization, and resembles the clinical scenario of sensory loss commonly existing alongside allodynia/hyperalgesia. Hence, we aimed to examine how spinal hyperexcitability and nonspinal mechanisms, namely, the dorsal column pathway that mainly innervates the VPL without many spinal terminations (Miki et al. 2000; Suzuki and Dickenson 2002), may converge onto thalamic relays, because this could aid in identifying the mechanisms and range of sensory abnormalities in SNL rats and the roles of WDR and NS neurons within this ascending pathway. Furthermore, excitability of thalamic neurons is influenced by cortical modulation (Monconduit et al. 2006), and thus the roles of WDR and NS neurons in the thalamus can be examined within the context of being subjected to “bottom-up” and “top-down” processing mechanisms.

In neuropathic conditions, it could be expected that if NS neurons gained sensitivity to low-threshold stimuli, i.e., became “WDR like,” that a disproportionate number of WDR neurons would be identified in neuropathic rats. We found little evidence for widespread changes in neuronal profiles. Likewise in the dorsal horn, sciatic nerve ligation does not induce changes in the frequency distribution of WDR and NS spinoparabrachial lamina I neurons (Andrew 2009), although it has been proposed that the stimulus-response dynamics of deeper dorsal horn spinothalamic NS neurons are altered after nerve injury whereas spinothalamic WDR firing frequencies are unaffected (Lavertu et al. 2014). Our data support the possibility that WDR neurons and not NS neurons encode hypersensitivity to low-threshold punctate mechanical and cool stimuli within this ascending channel. This conflicting conclusion with the latter study may arise from methodological differences in characterizing neuronal response profiles and from their reliance on correlations with spinal reflexes and the interpretation of the roles of WDR and NS neurons by considering only spino-bulbo processing mechanisms.

The broad changes in evoked hypersensitivity across intensities and modalities are consistent with a postsynaptic change in spinal neuronal excitability. GABAergic inhibition is reduced in the dorsal horn following a peripheral nerve injury (Moore et al. 2002); however, this does not appear to confer sensitivity to low-threshold stimuli to substantial numbers of thalamic NS neurons. Diminished GABAergic inhibition of spinal NS neurons may be largely restricted to gating of high-threshold input into the superficial dorsal horn. As a consequence, VP NS neurons retain the capacity to code to noxious stimuli in neuropathic conditions but with elevated firing frequencies. In the absence of frequency-dependent changes in firing at the spinal level and in the gracile nucleus, an expansion of neuronal receptive field sizes and the recruitment of increasing numbers of neurons could converge onto thalamic relays and underlie the elevated thalamic neuronal responses observed (Coghill et al. 1993; Suzuki and Dickenson 2002; Suzuki et al. 2000).

Brush and cold allodynia are frequent occurrences across a range of neuropathies and are a significant clinical challenge (Maier et al. 2010). Both WDR and NS neurons exhibit higher firing frequencies to brushing in SNL rats. Although low in number, we also find evidence that NS neurons may gain brush sensitivity in neuropathic conditions. This possibly reflects disinhibition at the spinal level, resulting in the opening of polysynaptic interneuronal pathways from deeper to more superficial laminae (Schoffnegger et al. 2008). Brush sensitivity of lamina I neurons in the dorsal horn is subjected to tonic glycinergic inhibition in normal rats (Miraucourt et al. 2007), and intrathecal strychnine block of glycine receptors can induce brush sensitivity in previously unresponsive NS neurons in the VPL (Sherman et al. 1997).

In neuropathic patients, cold temperatures can evoke sharp, stabbing sensations in addition to paradoxical burning, and likewise in SNL rats, cold hypersensitivity appears to be the major thermal disturbance. Cold hyperalgesia is hypothesized to result from spinal disinhibition, which subsequently converges on medial and lateral thalamic pathways, culminating in the unmasking of burning pain (Craig and Bushnell 1994; Ochoa and Yarnitsky 1994). Clinical evidence from stroke patients indicates that lesions to the ventralis caudalis (analogous to the VP in rats), but not extending to the VMpo (a postulated cold relay), are associated with a loss of cold sensitivity and hyperalgesia (Greenspan et al. 2004; Kim et al. 2007). Both increases in individual neuronal responses to cooling and increases in the number of responsive neurons are apparent in neuropathic rats. Under normal conditions a considerable component of cold input into the thalamus, and presumably the dorsal horn, is subthreshold, and at the spinal level, a loss of cross inhibition (McCoy et al. 2013) or an increase in postsynaptic excitability would be consistent with these observed changes in neuronal properties. In tandem, the clinical observations and electrophysiological evidence from the deep dorsal horn (Patel et al. 2015; Patel et al. 2014b) and VP supports the importance of the STT–VP–S1-S2 pathway to normal and aberrant cold sensitivity.

Increased spontaneous activity of spinal neurons has been reported following spinal nerve and sciatic nerve injury, typically with irregular patterns of firing (Palecek et al. 1992; Suzuki and Dickenson 2006). Interestingly, we find evidence of higher rates of spontaneous firing in WDR but not NS neurons in the VP. The intrinsic properties of WDR neurons may be altered, because VP neurons can express rapidly repriming Na_V_1.3 channels following spinal cord injury, contributing to an increase in basal neuronal excitability, and spinal knockdown of Na_v_1.3 attenuates these thalamic changes (Hains et al. 2005). Denervation of the thalamus can induce increased spontaneous activity (Weng et al. 2000), and a large part of ongoing activity may not reflect spinal neuronal activity (Fischer et al. 2009; Hains et al. 2005; Miki et al. 2000), although intrathecal lidocaine can produce conditioned place preference following nerve injury, and analgesic manipulations within the anterior cingulate cortex can block ongoing pain but fail to modulate evoked responses (He et al. 2012; Navratilova et al. 2015). Elevated spontaneous activity alters the dynamics within thalamocortical networks and coupling between cortical and thalamic structures (LeBlanc et al. 2016), and this thalamocortical dysrhythmia has been hypothesized to result in ongoing pain (Alshelh et al. 2016; Henderson et al. 2013). Neuronal correlates of ongoing pain would be of great importance to the back- and forward translation of therapeutics, and we have examined the effects of pregabalin within this context.

There is emerging evidence that a sensory profiling approach to stratify patients leads to better targeting of treatments including gabapentinoids (Baron et al. 2012; Freeman et al. 2014; Simpson et al. 2010), and this concept applied to animal models could aid translation. The antinociceptive activity of pregabalin is dependent on the interaction with the α_2_δ-1 subunit of calcium channels (Field et al. 2006; Patel et al. 2013), and the upregulation of α_2_δ-1 in the dorsal root ganglion and enhanced descending serotonergic facilitatory drive following nerve injury are critical mechanisms that determine the pathophysiological state-dependent inhibition of evoked hypersensitivity (Bauer et al. 2009; Bee and Dickenson 2008; Suzuki et al. 2005). Pregabalin was without inhibitory effect in sham rats but selectively inhibited neuronal responses at intensities evoking elevated responses in SNL rats, with the exception of cold stimuli. After pregabalin administration, responses of WDR neurons in SNL rats to brush, punctate mechanical, and noxious heat were comparable to baseline responses in sham rats, demonstrating that pregabalin normalizes hypersensitivity to these stimuli at this dose. The greater magnitude of inhibition of mechanically evoked responses compared with heat strongly resembles the effect of gabapentinoids on spinal lamina I and V neurons in neuropathic rats (Bee and Dickenson 2008; Donovan-Rodriguez et al. 2005). These neuronal responses correspond relatively well to quantitative sensory testing measures in a small cohort of neuropathic patients and in a human surrogate model of pain demonstrating a more pronounced reversal of ongoing pain, cold, brush, and pinprick allodynia compared with heat hyperalgesia (Attal et al. 1998; Dirks et al. 2002). One possibility is that the discordant effect against cold hypersensitivity is explained by differential mechanisms of acute and chronic pregabalin dosing regimens (reviewed by Patel and Dickenson, 2016). Cold hypersensitivity has been attributed to spinal disinhibition (Ochoa and Yarnitsky 1994), whereas descending serotonergic facilitatory influences are important in mediating mechanical and heat hypersensitivity and the effects of pregabalin (Bee and Dickenson 2008; Suzuki et al. 2005). This difference in underlying mechanisms likely determines the modality selective effects of pregabalin in SNL rats.

In contrast to effects on spontaneous spinal activity after SNL (Suzuki and Dickenson 2006), aberrant spontaneous firing of VP WDR neurons was not reduced by pregabalin, consistent with this aspect of thalamic excitability not being entirely dependent on spinal activity. In SNL rats, pregabalin reduces elevated spontaneous activity in the right central nucleus of the amygdala, which is associated with affective state such as fear and anxiety (Gonçalves and Dickenson 2012). Supraspinal effects of pregabalin might include modulation of cortico-limbic pathways that relate to affective dimensions of pain. Although not a neuropathic state, symptoms of central sensitization are evident in fibromyalgia, and in these patients reduction of posterior insula activity by pregabalin would be consistent with the aforementioned notion (Harris et al. 2013).

### 

#### Conclusion.

These data reveal novel features of thalamic neuronal hyperexcitability in SNL rats. Pregabalin normalized neuronal hyperexcitability to mechanical and heat stimuli following neuropathy but lacked effect on elevated spontaneous activity or normal neuronal coding. These findings correlate with observations that gabapentinoids have a high NNT when ongoing pain is used as a primary endpoint in clinical trials (Finnerup et al. 2015; Moore et al. 2009) but improved efficacy in patient subgroups where mechanical hyperalgesia is prominent (Simpson et al. 2010). These neural substrates provide an opportunity to examine the effects of analgesics on integrated sensory processing within the brain.

## GRANTS

This study was funded by Wellcome Trust Pain Consortium Project 102645: Defining pain circuitry in health and disease.

## DISCLOSURES

No conflicts of interest, financial or otherwise, are declared by the authors.

## AUTHOR CONTRIBUTIONS

R.P. and A.H.D. conception and design of research; R.P. performed experiments; R.P. analyzed data; R.P. and A.H.D. interpreted results of experiments; R.P. prepared figures; R.P. drafted manuscript; R.P. and A.H.D. edited and revised manuscript; R.P. and A.H.D. approved final version of manuscript.
